# A predictive model of overall survival in patients with metastatic castration-resistant prostate cancer

**DOI:** 10.12688/f1000research.8192.2

**Published:** 2019-05-17

**Authors:** Mehrad Mahmoudian, Fatemeh Seyednasrollah, Liisa Koivu, Outi Hirvonen, Sirkku Jyrkkiö, Laura L. Elo

**Affiliations:** 1 Turku Centre for Biotechnology, Turku, Finland; 2 Department of Information Technology, University of Turku, Turku, Finland; 3 Department of Oncology and Radiotherapy, University of Turku, Turku, Finland; 4 Department of Clinical Oncology, University of Turku, Turku, Finland

**Keywords:** prostate cancer, mCRPC, boosting, survival analysis

## Abstract

Metastatic castration resistant prostate cancer (mCRPC) is one of the most common cancers with a poor prognosis. To improve prognostic models of mCRPC, the Dialogue for Reverse Engineering Assessments and Methods (DREAM) Consortium organized a crowdsourced competition known as the Prostate Cancer DREAM Challenge. In the competition, data from four phase III clinical trials were utilized. A total of 1600 patients’ clinical information across three of the trials was used to generate prognostic models, whereas one of the datasets (313 patients) was held out for blinded validation. The previously introduced prognostic model of overall survival of chemotherapy-naive mCRPC patients treated with docetaxel or prednisone (so called Halabi model) was used as a performance baseline. This paper presents the model developed by the team TYTDreamChallenge and its improved version to predict the prognosis of mCRPC patients within the first 30 months after starting the treatment based on available clinical features of each patient. In particular, by replacing our original larger set of eleven features with a smaller more carefully selected set of only five features the prediction performance on the independent validation cohort increased up to 5.4 percent. While the original TYTDreamChallenge model (iAUC=0.748) performed similarly as the performance-baseline model developed by Halabi et al. (iAUC=0.743), the improved post-challenge model (iAUC=0.779) showed markedly improved performance by using only PSA, ALP, AST, HB, and LESIONS as features. This highlights the importance of the selection of the clinical features when developing the predictive models.

## Introduction

Prostate cancer is the second most common cancer according to the World Cancer Report 2014 ^1^. Hence it is one of the most studied cancer types with focus on diagnosis and prognosis. A major cause of death among prostate cancer patients is the development of metastatic castrate-resistant prostate cancer (mCRPC), which is both a persistent as well as progressing disease resistant to androgen deprivation therapy ^2^.

In order to boost research regarding prostate cancer, a crowdsourced competition was designed by the Dialogue for Reverse Engineering Assessments and Methods (DREAM) Consortium in collaboration with *Project Data Sphere LLC* (PDS) to improve prognostic models of mCRPC. Using data from four phase III clinical trials available through PDS, two main sub-challenges were designed. Sub-challenge 1 was aimed at improving prediction of survival risk for mCRPC patients, whereas Sub-challenge 2 was intended to predict adverse events in patients treated with docetaxel, the standard of care for mCRPC patients at the time of the trials. This paper presents the model developed by the team TYTDreamChallenge for the Sub-challenge 1 to predict survival risk scores for mCRPC at 12, 18, 24 and 30 months after diagnosis based on clinical features of each patient, as well as a post-challenge analysis to improve our initial model.

Various prognostic models for mCRPC have been previously developed ^3– 5^. Recently, Halabi *et al*. developed a prognostic model for mCRPC using eight clinical features (Eastern Cooperative Oncology Group performance status, disease site, lactate dehydrogenase, opioid analgesic use, albumin, hemoglobin, prostate-specific antigen, and alkaline phosphatase) and validated it on an external dataset. We refer to this model as Halabi model. It was used in the DREAM Challenge as a performance-baseline model.

In the TYTDreamChallenge model and in our post-challenge model, we used generalized boosted models implemented in the R ^6^ package gbm ( generalized boosted regression models) to predict overall survival of mCRPC patients with Cox proportional hazard model as the underlying regression model ^7^. The gbm package is an extension to the Freund and Schapire's AdaBoost algorithm ^8^ and Friedman's gradient boosting machine ^9^. In general, boosting is a concept in supervised machine learning with the goal of generating multiple relatively weak learner models, which each individually work slightly better than random guess, and use them all in corporation to have a highly accurate overall model ^10^.

## Methods

Our methodology consisted of two major steps ^11^. The first step was data preparation. The second step was model building utilizing generalized boosted models. All the validations of the model predictions were performed through the submission system of the DREAM Challenge, where the true response values in the validation data remained hidden.

### Data

The data used in this study was collected from mCRPC patients by four institutes. The datasets were based on a cancer treatment trial in which patients received docetaxel treatment. Details of the four trials are shown in Table 1. In the Prostate Cancer DREAM Challenge, three (ASCENT-2 ^12^, MAINSAIL ^13^ and VENICE ^14^) out of the four datasets were available as training sets. The remaining dataset (ENTHUSE-33 ^15^) was used for validation by the DREAM Challenge organizers without releasing the survival data to the participants of the competition. All the data were gathered into five major tables ( Supplementary Table 1). Additionally, a sixth table, called CoreTable, was provided by the challenge organizers. The CoreTable was a collection of features from the other five tables that summarized the baseline (day 0) values. The clinical features in CoreTable contained treatment variables, cancer staging based on AJCC ^16^, Gleason Score ^17^, ECOG Performance Status ^18^, and lesion details. This table was curated by challenge organizers and was considered as the main table in the Challenge.

**Table 1.** The four clinical trial datasets used for the mCRPC predictions.

Data Provider	ID	Number of patients	Reference
**Novacea, provided by Memorial** **Sloan Kettering Cancer Center**	ASCENT-2	476	Scher *et al*. ^13^
**Celgene**	MAINSAIL	526	Petrylak *et al*. ^14^
**Sanofi**	VENICE	598	Tannock *et al*. ^15^
**AstraZeneca**	ENTHUSE-33	470	Fizazi *et al*. ^16^

Out of all the data provided, we focused on the CoreTable and LabValue tables to form the training and validation datasets. The LabValue table was an event level longitudinal data table which contained all the lab tests performed along with the sampling date and reference range of each lab test. The CoreTable consisted of 131 features, of which two were for identification, five were dependent variables and 124 were independent variables. The two dependent variables we used in this study were DEATH and LKADT_P. The variable DEATH indicates the death status of a patient and has value “YES” for patients who died from mCRPC and value “NO” otherwise. The variable LKADT_P is the last day that the patient was known to be alive.

The full set of the Challenge data is available under the standard Synapse Terms and Conditions of Use and the Prostate Cancer DREAM Challenge Rules and can be downloaded from Synapse web interface. The links and authentication information are available in the following URL: https://www.synapse.org/ProstateCancerChallenge

### Data preparation

Processing of the laboratory values (table LabValue) consisted of a sequence of actions. First, it was observed that there were some duplicate rows in the data; hence 2545 rows were removed. Secondly, based on consultation with oncologists, rows with measurements of 13 lab tests were extracted, including ALT, AST, ALP, LDH, MG, PHOS, ALB, TPRO, PSA, HB, WBC, NEU and LYM ( Table 2). After this step, the number of rows left in the data was 80744. Thirdly, we removed 603 rows marked with “NOT DONE” status in the LBSTAT column, which specifies completion status of the lab test, and with missing value in their LBSTRESC column, which contains standardized format of the test results. Finally, only the 17015 baseline measurements from the 1599 patients were kept in the study, while removing the other follow-up measurements over time as they were unavailable in the validation data. During the steps explained above, one patient (ASC-518-0003) was completely removed from the analysis because of having “NOT DONE” status in most of the lab tests, including ALT, AST, ALP and LDH.

**Table 2.** Definitions of the 13 lab tests selected on the basis of consultation with oncologists.

Lab Test Abbreviation	Definition
**ALT**	Alanine aminotransferase
**AST**	Aspartate aminotransferase
**ALP**	Alkaline phosphatase
**LDH**	Lactate dehydrogenase
**MG**	Magnesium
**PHOS**	Phosphorus
**ALB**	Albumin
**TPRO**	Total protein
**PSA**	Prostate specific antigen
**HB**	Hemoglobin
**WBC**	White blood cells
**NEU**	Neutrophils
**LYM**	Lymphocytes

The measurement values for all lab tests, except PSA, were standardized based on their minimum and maximum ranges as

$$x^{\prime }=2\cdot \frac{x-\alpha }{\beta -\alpha }-1\;(1)$$

where *x* is the observed value of the lab test and *β* and *α* are the corresponding upper and lower limit of the reference range. The standardized values are between -1 and 1 if the lab test value is within the normal range. The PSA values were only log _2_ transformed and the issue of log _2_0 was bypassed by adding e- ^4^ to the values before log _2_ transformation. The ALP and NEU values were truncated to 10 and 5, respectively.

In the validation dataset, there were two patients (AZ-00131 and AZ-00383) that had no records in the LabValue table nor in the CoreTable. To predict their survival using the laboratory values, we extracted medians of the 13 lab test features across all patients and used them for these two patients.

In addition to lab tests, we considered some additional features from the CoreTable. These included ECOG_C and ANALGESICS as well as four derived features that were summarized to reduce the variation and existing noise in the data. These included LESIONS, DRUGS, DISEASES and PROCEDURES, which were defined as arithmetic sums of the numbers of lesions, medicines, diseases or medical operations, respectively. LKADT_P and DEATH were also directly adopted from the CoreTable.

**Supplementary Figure 1.**
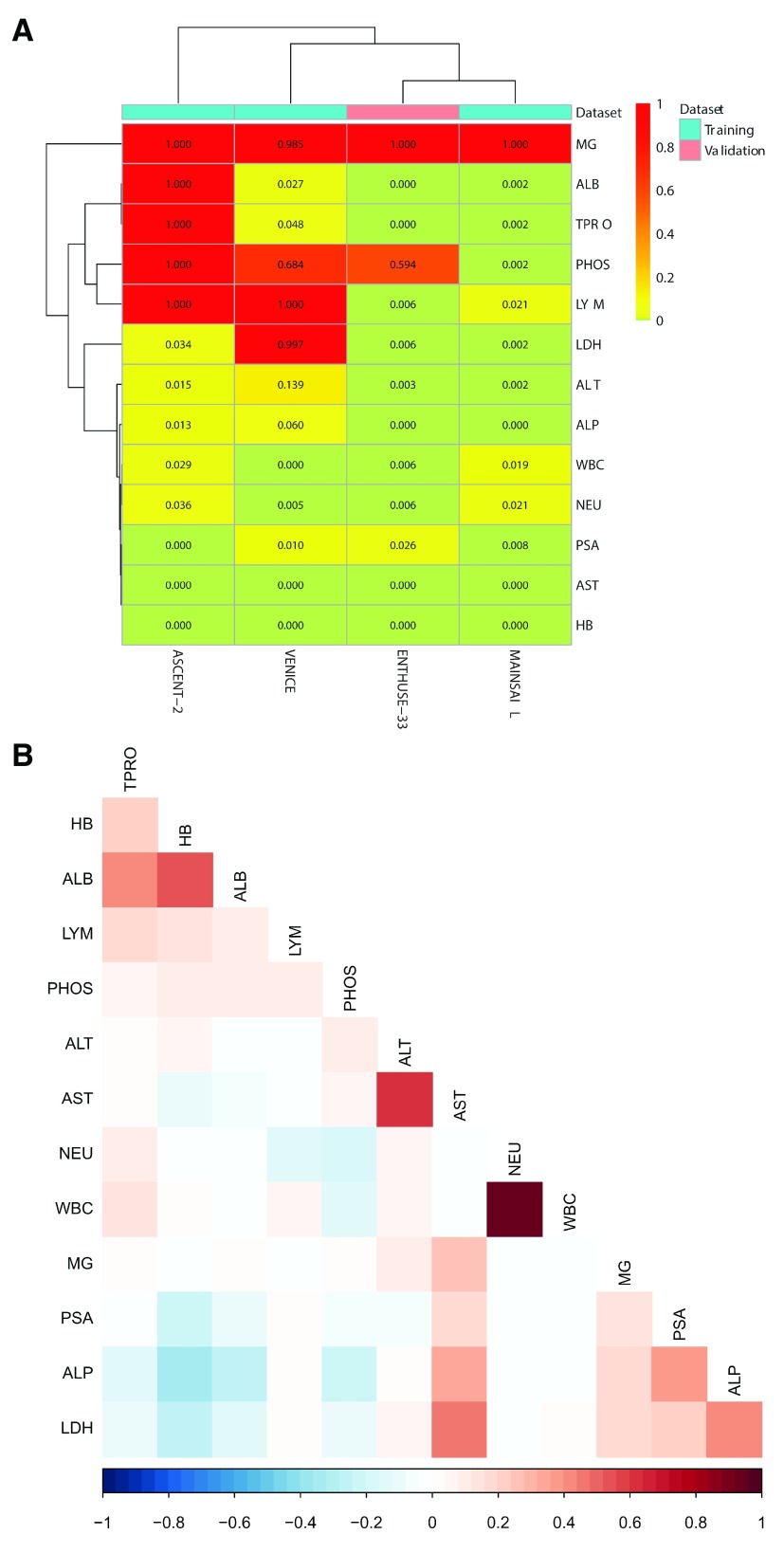
Missing values and correlations in the training data. ( **A**) Proportions of missing values for the 13 lab tests selected on the basis of consultation with oncologists. The proportion of missing values is shown separately for each clinical trial dataset (columns), with red indicating large proportions of missing values. ( **B**) Pearson correlation between the different features across all studies. The darker the color, the higher the absolute value of correlation.

As the final step in pre-processing, the resulting training and validation datasets were checked for features having large proportions of missing values or having missing values for a particular data provider. The missingness in data is shown in Supplementary Figure 1A. Based on this, six features including MG, ALB, TPRO, LYM, PHOS, and LDH were excluded from the training and validation sets with maximum missingness of ~100% in at least one of the datasets. Additionally, to minimize the number of highly correlated features in the training data, we further removed WBC and ALT, which showed high correlation with NEU and AST, respectively ( Supplementary Figure 1B).

At the end of the pre-processing, the training set consisted of 1599 patients and validation set of 313 patients. Both datasets had 15 features out of which two were for identification, two were response variables and the other 11 were independent predictor variables (ECOG_C, ANALGESICS, LESIONS, DRUGS, DISEASES, PROCEDURES, AST, ALP, PSA, HB, and NEU).

### Machine learning and survival prediction

To develop a model of overall survival in mCRPC, we utilized a gradient boosting algorithm based on regression trees, with a Cox proportional hazard model as the underlying regression model. The R package gbm ^19^ was used with 5000 trees, 10-fold cross-validation, minimum 3 observations in the trees’ terminal nodes, and step-size reduction value of 0.007.

In the DREAM Challenge competition, we considered a separate risk score for 12, 18, 24 and 30 months. For 18, 24 and 30 months, we built a separate model for each data provider, and the mean of the three individual risk score predictions was calculated as the final risk score at each time point. For 12 months, all the training data were used to create a single model and risk score prediction. After the challenge, we also tested these two strategies in constructing a single overall risk score for each patient: 1) average of risk scores obtained separately for each data provider (referred to as PostSeparate), or 2) a single risk score obtained by combining data from all the providers in the modelling (referred to as PostCombined).

### Performance evaluation

The performance of the predictions was measured using the integrated area under the ROC curve (iAUC) from 6 to 30 months, as well as separate AUC values at 12, 18, and 24 months. The iAUC was calculated using the R package timeROC (version 0.3) ^20^. The performance measures were obtained from blinded validation by the DREAM Challenge organizers.

## Results and discussion

The performance of the TYTDreamChallenge model (iAUC=0.748) was significantly better than random. However, it did not perform statistically significantly better than the performance baseline Halabi model (iAUC=0.743, Bayes factor < 3), as determined by the DREAM Challenge organizers ^21^.

**Figure 1.**
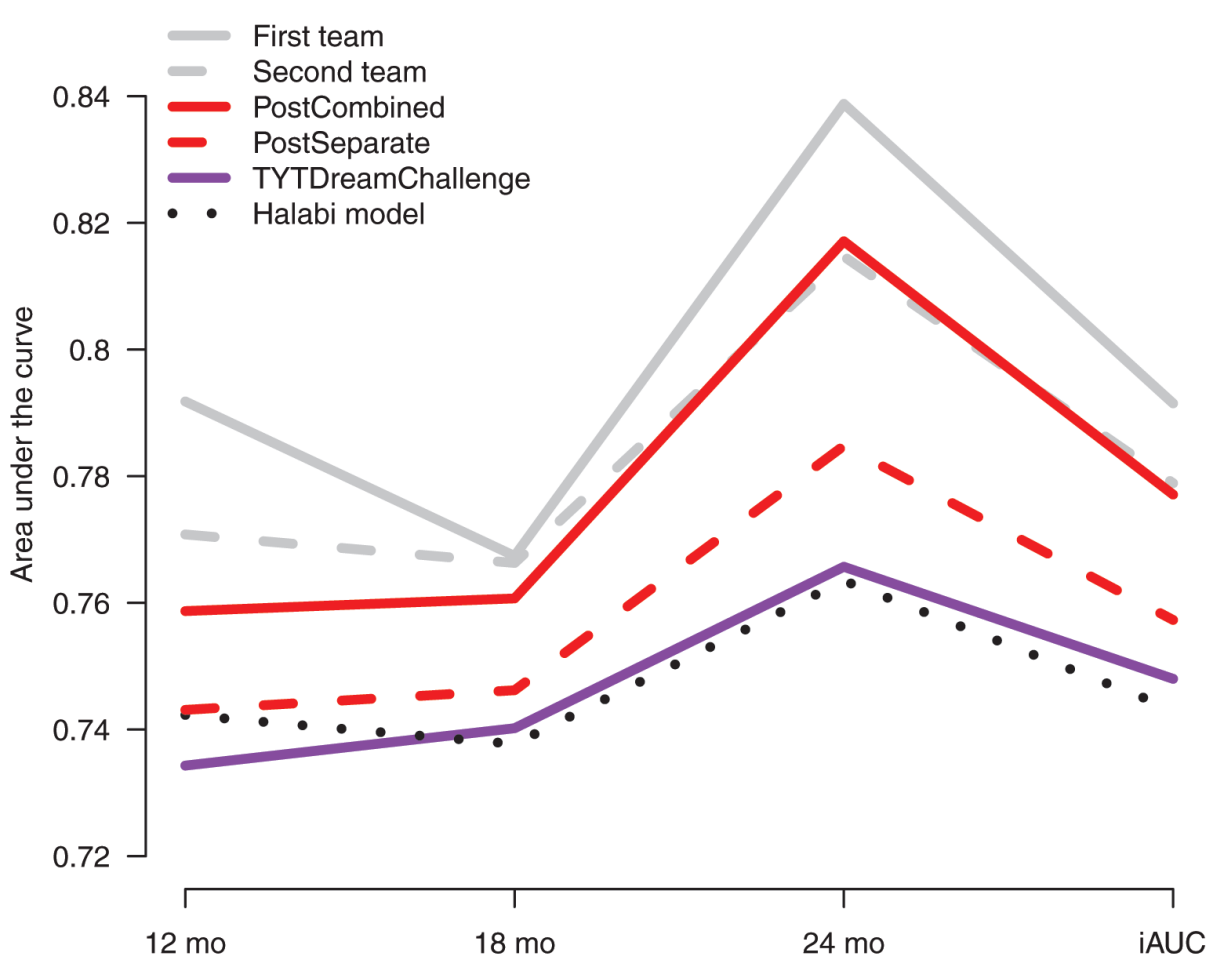
Performance of the different models compared to the performance baseline Halabi model. The integrated area under the ROC curve (iAUC) from 6 to 30 months, as well as separate AUC values at 12, 18, and 24 months are shown. The performance measures were obtained from blinded validation by the DREAM organizers. The first and second team refer to the top-ranked models in the DREAM Challenge; PostCombined and PostSeparate refer to two different post-challenge analyses using the same modelling strategy and same features as in our original DREAM Challenge submission (TYTDreamChallenge) but, instead of having time-specific models, a single overall risk score was calculated for each patient either as an average risk score across the data providers (PostSeparate) or as a single risk score obtained by combining data from all the providers in the modelling (PostCombined).

To further investigate the possibility to improve our model after the challenge, we considered in our post-challenge analysis the impact of calculating an overall risk score instead of our original strategy of having separate scores for the different time points. Interestingly, this had a marked effect on the performance of our model ( Figure 1). When the average model across the different data providers was considered, the iAUC improved to 0.757 (model PostSeparate). When all the data were used together for model building, the iAUC increased further to 0.777 (model PostCombined).

**Figure 2.**
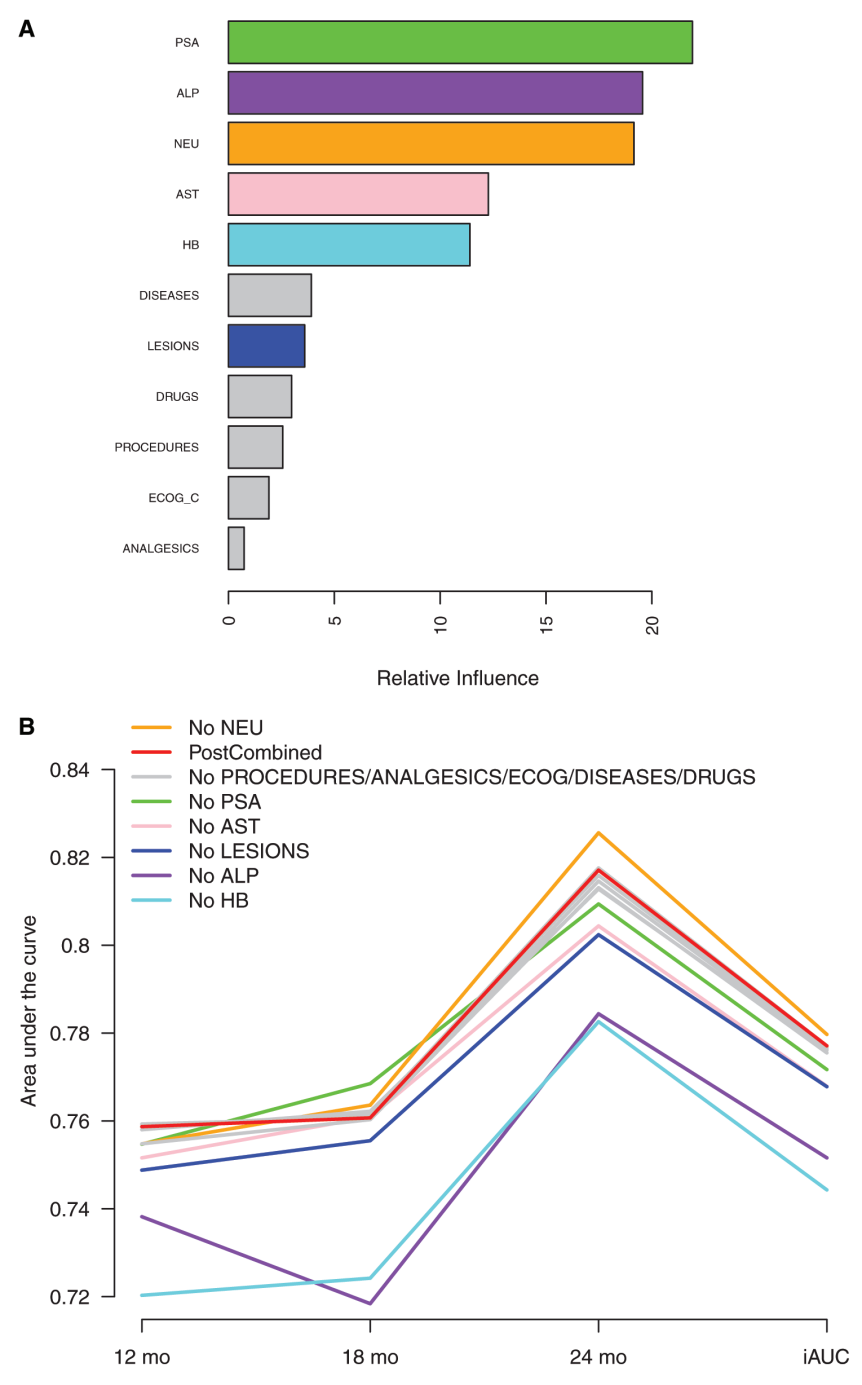
Relative importance of the different features on the predictions. ( **A**) Relative influence in the post-challenge model where a single overall risk score was calculated for each patient by combining data from all the providers in the modelling (PostCombined). ( **B**) Effect of removing one feature at a time when building the model. The integrated area under the ROC curve (iAUC) from 6 to 30 months, as well as separate AUC values at 12, 18, and 24 months are shown. The performance measures were obtained from blinded validation by the DREAM organizers.

Next, we examined the relative importance of the different features on the predictions in the PostCombined model, as determined by the boosting algorithm ( Figure 2A). As expected, many of the features used in the Halabi model had high importance also in our model (PSA, ALP, and HB). However, additional features were found (AST, NEU). On the other hand, ECOG_C was not as important in our model as it was in the Halabi model. We also tested the effect of removing one variable at a time when building the model ( Figure 2B). This supported further the importance of ALP, HB, AST, PSA and LESIONS, whereas the removal of NEU actually improved the performance further (iAUC=0.780). Removal of PROCEDURES, ANALGESICS, ECOG_C, DISEASES or DRUGS did not have a marked impact on the performance.

**Figure 3.**
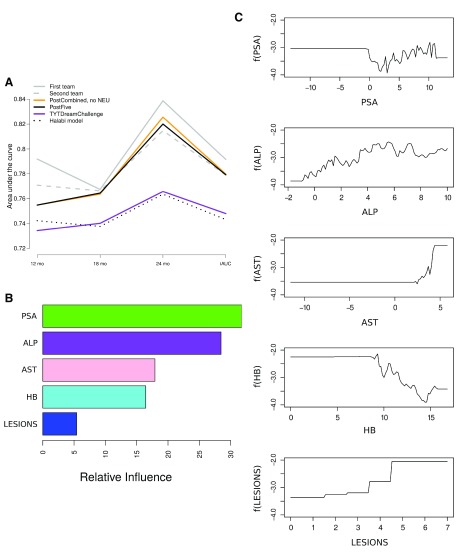
PostFive model. ( **A**) Performance of the boosting strategy using only five features ALP, HB, LESIONS, AST and PSA, as compared to the DREAM Challenge models and our post-challenge models. The integrated area under the ROC curve (iAUC) from 6 to 30 months, as well as separate AUC values at 12, 18, and 24 months are shown. The performance measures were obtained from blinded validation by the DREAM organizers. ( **B**) Relative importance of the different features on the predictions. ( **C**) Partial dependence plots illustrating the marginal effect of changing the value of each feature (x-axis) on the value of the hazard function (y-axis), while averaging out the other variables. In general, higher hazard value can be interpreted as lower survival probability.

Finally, we applied the same boosting strategy to build a model using only five features ALP, HB, LESIONS, AST and PSA ( Figure 3A; referred to as PostFive). Notably, the performance in the validation data did not decrease markedly from that with a larger set of features (iAUC=0.779). Among the features, PSA and ALP had the largest relative importance in predicting the survival, whereas LESIONS had the lowest relative importance ( Figure 3B). To assist in understanding the contribution of the identified features, partial dependence plots were examined, which illustrate the partial dependence of the risk scores on each feature after accounting for the effects of the other features ( Figure 3C). Similarly as in the Halabi model, the risk increases with high values of PSA and ALP, high numbers of LESIONS, and low values of HB ^22^. Additionally, our model suggests that high values of AST increase the risk. These findings are well in line with the general hypothesis that these factors are basic values that associate with patients’ prognosis.

Taken together, based on the blinded validations it can be concluded that the proposed post-challenge model in this paper (PostCombined) was markedly better than the Halabi model, which is considered as the state-of-the-art standard method in the field. The post-challenge analysis suggested that a single overall risk score performed better than our original strategy of time-specific risk scores by better targeting the overall survival pattern of patients. A model based on only five features ALP, HB, AST, PSA and LESIONS produced a relatively high accuracy compared to the Halabi model with eight features or the model of the winning team involving a large number of features and their interactions. Thus the five-feature model (PostFive) presented here provides an efficient option in terms of practical clinical use.

The present study focused on clinical features only. Additional possibilities to improve the performance of the models would be to add molecular level information, such as gene expression data to training and test sets.

## Data and software availability

The Challenge datasets can be accessed at: https://www.projectdatasphere.org/projectdatasphere/html/pcdc

Challenge documentation, including the detailed description of the Challenge design, overall results, scoring scripts, and the clinical trials data dictionary can be found at: https://www.synapse.org/ProstateCancerChallenge

The code and documentation underlying the method presented in this paper can be found at: http://dx.doi.org/10.5281/zenodo.47706 ^23^

The latest source code is available at: https://bitbucket.org/mehrad_mahmoudian/dream-prostate-cancer-challenge-q.1a

## Figures and Tables

**Supplementary Table 1.** Raw data available in the Challenge.

Table name	Table description
**PriorMed**	Prior Medication table records the medication that the patients have taken before the first treatment date of the trial.
**MedHistory**	Medical History table records the diagnoses reported by the patients (co-existing diseases) at the time of patient screening to participate in the trial.
**LesionMeasure**	Lesion table records target and non-target lesion measurements.
**LabValue**	Lab test table includes all data from lab tests (hematology and urinary lab).
**VitalSign**	Vital Sign table records vital signs of the patients (e.g. height, weight, etc.).
