# Supplementary material for: A predictive model of overall survival in patients with metastatic castration-resistant prostate cancer
Source: F1000Res. 2019 May 17;5:2674. Originally published 2016 Nov 16. [Version 2] doi: 10.12688/f1000research.8192.2 (PMC6556990; doi:10.12688/f1000research.8192.2)
Supplement: Peer review history

# Peer review: Reviewer response for version 2

**DOI:** 10.5256/f1000research.20848.r48714

**Published:** 2019-06-04

**Authors:** Peter K Rogan

**Affiliations:** 1 Dept of Biochemistry, Schulich School of Medicine and Dentistry, University of Western Ontario, London, ON, N6A 5C1, Canada

I approve the revised article based on the revisions made by the authors.

I have read this submission. I believe that I have an appropriate level of expertise to confirm that it is of an acceptable scientific standard.

# Peer review: Reviewer response for version 1

**DOI:** 10.5256/f1000research.8812.r23094

**Published:** 2017-06-26

**Authors:** Peter K Rogan

**Affiliations:** 1 Dept of Biochemistry, Schulich School of Medicine and Dentistry, University of Western Ontario, London, ON, N6A 5C1, Canada

In general, the paper is understandable and well organized. The use of boosting to improve the Cox proportional hazard and regression models is interesting. Despite the issues the authors address regarding missing data, there appears to be adequate information in this dataset to test this approach.

**Abstract**

 “as well as an improvement of the model developed after the challenge”

 This is vague statement that adds little to the abstract. It is irrelevant when the improvement was made. The abstract should describe the clinical features and any improvements in an organized manner so that the reader can determine whether the method proposed differs from previous approaches.

 “TYTDreamChallenge model performed similarly as the gold-standard Halabi model”

 What was the performance accuracy or misclassification rate?

 The “Halabi” model terminology is jargonistic and should be described briefly in conventional oncology criteria (ie. an abbreviated form of the statement in the introduction would be sufficient). Was the increase in the ROC by 3% significant? If so, please explain why.

**Introduction**

 The authors term the paper by Halabi *et al.* to be the “gold standard” prognostic model. While it performs well and is a reasonable comparator, it does not meet the criteria for a gold standard. It is a conventionally trained and tested and validated with a single external dataset. Gold standards, on the other hand, have been reproduced by other investigators using other patient cohorts multiple times with similar findings, may have fulfilled ISO or other standards and have been recommended by internationally recognized authorities (for example, the dicentric chromosome assay for radiation dosimetry).

**Methods**

 “some features were removed from the study due to missing values, high correlations with other features or being unimportant for such survival analysis as determined by clinical experts. “

 Please indicate which features were removed. What proportions were attributable to missing values, etc.

 “based on consultation with oncologists, rows with measurements of 13 important lab tests”

 Define important vs unimportant lab tests, and reasons for selecting them.

 Did the authors evaluate whether their methods were  very sensitive to assumptions made about the missingness mechanism or about the distributions of the variables with missing data? If so, please state.

**Results**

 The authors don’t clearly distinguish which methods were used in their submission of results to the DREAM challenge vs. how or why the “improvements” were made after the submission to the challenge. Furthermore, the overall risk score vs separate scores at different time points can simply be reported, without making this artificial distinction. Thus, the distinction made in the abstract between these lacks context, and I would recommend removing it.

 There are many other measures for evaluating the models that the authors could report besides AUC, including Matthews Correlation Coefficient, F-measure, Precision, and Accuracy. They may consider computing and reporting these.

 Figure 3 is unacceptable quality. Even at 200% magnification, the labels on each of the graphs are barely readable. Panel C requires some further explanation in order to interpret it.  The text indicates “This suggested intuitive interpretations for the different features,” which does not explain the results or whether the partial dependence can be used for feature selection or interpretation.

 “These findings are well in line with the general hypothesis that these factors are basic values representing the volume of the disease.”

 By volume, are the authors referring to the extent of the disease? The extent of the disease is not the same as the survival risk, which is what the authors state they are modeling in the introduction.   In cancer, volume refers to the size of the tumor and quantitative distribution (Castro-Mesta *et al.* 2016 ^1^). In fact, the authors use the numbers of lesions to infer survival risk, so it would appear that this is a circular argument.

I have read this submission. I believe that I have an appropriate level of expertise to confirm that it is of an acceptable scientific standard, however I have significant reservations, as outlined above.

## References

[1] Castro-MestaJGonzález-GuerreroJBarrios-SánchezPVillarreal-CavazosG: Bases and foundations of the treatment of peritoneal carcinomatosis: Review article. *Medicina Universitaria*.2016;18(71) : 10.1016/j.rmu.2016.06.001 98-104 10.1016/j.rmu.2016.06.001

# Response

**Published:** 2019-04-23

**Authors:** Mehrad Mahmoudian

**Affiliations:** Turku Centre for Biotechnology, Finland

We would like to thank the reviewer for the time and effort he has put into our manuscript and the insightful comments. We have addressed all the comments in this response as well as in the main text and figures accordingly.

**Abstract**

*> “as well as an improvement of the model developed after the challenge”*

*> This is vague statement that adds little to the abstract. It is irrelevant when the improvement was made. The abstract should describe the clinical features and any improvements in an organized manner so that the reader can determine whether the method proposed differs from previous approaches.*

 Thank you for your comment. We understand your concern that the text was not clear enough to convey the concept we wanted to the full extent. Therefore, we have modified the text to improve clarity and transparency and included information about the clinical features in the abstract as suggested by the reviewer. Regarding the time of analysis, we feel that it should be mentioned to comply with the DREAM Challenge procedure and evolution of developing the ultimate model.

*> “TYTDreamChallenge model performed similarly as the gold-standard Halabi model”*

*> What was the performance accuracy or misclassification rate?*

 All the validations of the predictions were done through submitting the predicted values to the DREAM Challenge evaluation system as have been explained in detail in https://www.synapse.org/#!Synapse:syn2813558/wiki/209591 . As the true response values of the validation dataset were kept hidden from the participants, unfortunately, the requested information cannot be provided. We have modified the text to clarify this fact accordingly.

*> The “Halabi” model terminology is jargonistic and should be described briefly in conventional oncology criteria (ie. an abbreviated form of the statement in the introduction would be sufficient). Was the increase in the ROC by 3% significant? If so, please explain why.*

 We have clarified the term “Halabi model” in the abstract via:

 “The previously introduced prognostic model of overall survival of chemotherapy-naive mCRPC patients treated with docetaxel or prednisone (so called Halabi model) was used as a performance baseline.”

 Regarding the comment about significance, unfortunately as explained above, we do not have access to the true response values and therefore the significance between the ROC curves cannot be computed.

**Introduction**

*> The authors term the paper by Halabi et al. to be the “gold standard” prognostic model. While it performs well and is a reasonable comparator, it does not meet the criteria for a gold standard. It is a conventionally trained and tested and validated with a single external dataset. Gold standards, on the other hand, have been reproduced by other investigators using other patient cohorts multiple times with similar findings, may have fulfilled ISO or other standards and have been recommended by internationally recognized authorities (for example, the dicentric chromosome assay for radiation dosimetry).*

 We agree that the term “gold-standard” was not a good choice. Therefore, we have changed all instances to “performance baseline”.

**Methods**

*> “some features were removed from the study due to missing values, high correlations with other features or being unimportant for such survival analysis as determined by clinical experts. “*

*> Please indicate which features were removed. What proportions were attributable to missing values, etc.*

 We agree that the description of the feature selection process was not very clear. We have now modified the text. The revised Supplementary Figure 1 now shows the proportions of missing values and the correlations between the 13 lab tests considered. We also provide the source codes of this study publicly available, containing the in-depth details of the feature exclusion along with the exact procedure to reproduce the results. The DOI of the published code is doi:10.5281/zenodo.47706 which is also presented as the last reference of the manuscript.

*> “based on consultation with oncologists, rows with measurements of 13 important lab tests”*

*> Define important vs unimportant lab tests, and reasons for selecting them.*

 “Important” referred to the educated estimation of the oncologists about the importance of investigating these features. We have now rephrased the text.

*> Did the authors evaluate whether their methods were very sensitive to assumptions made about the missingness mechanism*

 The features used in the modelling contained only relatively few missing values. Therefore, in the current study, we did not investigate the missingness mechanisms.

**Results**

*> The authors don’t clearly distinguish which methods were used in their submission of results to the DREAM challenge vs. how or why the “improvements” were made after the submission to the challenge. Furthermore, the overall risk score vs separate scores at different time points can simply be reported, without making this artificial distinction. Thus, the distinction made in the abstract between these lacks context, and I would recommend removing it.*

 The main difference between the original submission and the post-challenge submission was a more systematic feature selection approach in the latter. This is now clarified in the revised manuscript. According to the suggestion by the Reviewer, we have now removed from the abstract the distinction between the overall risk score vs separate scores at different time points.

*> There are many other measures for evaluating the models that the authors could report besides AUC, including Matthews Correlation Coefficient, F-measure, Precision, and Accuracy. They may consider computing and reporting these.*

 The true response values of the independent validation set are not publicly available. Therefore, it is not possible to calculate other performance measures than those available from the DREAM evaluation system.

*> Figure 3 is unacceptable quality. Even at 200% magnification, the labels on each of the graphs are barely readable.*

 We agree that the font size could be larger and therefore we have increased the font size in Figure 3.

*> Panel C requires some further explanation in order to interpret it. The text indicates “This suggested intuitive interpretations for the different features,” which does not explain the results or whether the partial dependence can be used for feature selection or interpretation.*

 We agree that the legend did not address the interpretation of the partial dependence plots. Therefore, we have completely revised the text for panel C and have elaborated on the interpretation.

*> “These findings are well in line with the general hypothesis that these factors are basic values representing the volume of the disease.”*

*> By volume, are the authors referring to the extent of the disease? The extent of the disease is not the same as the survival risk, which is what the authors state they are modeling in the introduction. In cancer, volume refers to the size of the tumor and quantitative distribution (Castro-Mesta et al. 2016* *1* *). In fact, the authors use the numbers of lesions to infer survival risk, so it would appear that this is a circular argument.*

 We agree with the reviewer that the term “volume” was not correct in this sentence. We have modified the sentence accordingly.

# Peer review: Reviewer response for version 1

**DOI:** 10.5256/f1000research.8812.r23226

**Published:** 2017-06-26

**Authors:** Motoki Shiga

**Affiliations:** 1 Department of Electrical, Electronic and Computer Engineering, Gifu University, Gifu, Japan

This paper offers a survival time prediction method based on a gradient boosting algorithm for a Cox proportional hazard model. The analysis of the Prostate Cancer DREAM Challenge dataset found that the prediction performance of a model built from all three clinical trials is much better than the performance of models built from each clinical trial.

**Major comments:**

 Additional comparison with the prediction performance of the Halabi model built from all clinical trials and the performance of the models built using each trial should help to evaluate method PostCombined in more detail. And the description of procedures of the first and second winning teams is cessary.

 What is the test data to compute iAUC? It was clinical trial AstraZeneca in the challenge. The manuscript should describe the procedure of the performance evaluation in detail.

**Minor comments:**

 Please explain the machine learning method used in this work in more detail.

 The threshold value of the feature selection should be described in the third paragraph of the right column in page 4.

I have read this submission. I believe that I have an appropriate level of expertise to confirm that it is of an acceptable scientific standard.
